# CHILDHOOD SES IS ASSOCIATED WITH FASTER WORSENING METABOLIC SYNDROME SEVERITY FOR BLACK RELATIVE TO WHITE WOMEN

**DOI:** 10.1093/geroni/igac059.691

**Published:** 2022-12-20

**Authors:** Agus Surachman, Nancy Adler, Barbara Laraia, Elissa Epel

**Affiliations:** University of California, San Francisco, San Francisco, California, United States; University of California, San Francisco, San Francisco, California, United States; University of California Berkeley, Berkeley, California, United States; University of California, San Francisco, San Francisco, California, United States

## Abstract

This analysis examined whether early life SES was associated with longitudinal changes in metabolic syndrome (MetS) severity among Black and white women. Data were from 531 women (non-Hispanic Black=263; non-Hispanic white=268, Mage=39) in the National Growth and Health Study (NGHS). Information about parental education was collected during the baseline survey when participants were 9. Information regarding MetS severity were collected during year-7 (Mage=16), year-10 (Mage=19), and year-30 (Mage = 39) follow-up studies. Controlling for baseline body mass index, smoking status, and marital status, Black participants showed a faster increase in MetS severity across two decades. Early life SES (b=.03, SE=.01, p<.05), independent of current SES, was associated with faster worsening MetS severity among Black relative to white women. The socioeconomic context of early rearing is an important factor for racial disparities in accelerated aging among these early midlife Black and white women.

